# Applying Human Factors Engineering Methods for Risk Assessment of a Neonatal Incubator

**DOI:** 10.1155/2019/8589727

**Published:** 2019-01-06

**Authors:** Renata Aparecida Ribeiro Custódio, Clarissa Trzesniak, Renata Pinto Ribeiro Miranda, Guilherme Henrique Donda Angelini, Jade Souza Bordón, Leila Cristina Santos Vieira, Carlos Henrique Pereira Mello

**Affiliations:** ^1^Federal University of Itajubá, Itajubá, MG 37500-903, Brazil; ^2^School of Medicine of Itajubá, Itajubá, MG 37502-138, Brazil; ^3^Clinical Hospital Samuel Libânio, Pouso Alegre, MG 37550-000, Brazil

## Abstract

This research aimed to find main users, frequent utilized tasks, major usability problems, and the context of use of a neonatal incubator (NI) present in a neonatal intensive care unit from a Brazilian hospital and to find out the problems faced by a new user. The chosen methods were the heuristics analysis, contextual investigation, and usability test (UT). Nurses and technicians are the main users of NIs. The predominant contexts of use are the admission of newborns and the replacement of the equipment. Eight selected tasks were performed in the UT, and the most significant problems refer to alarms and configuration of the Air and Skin Modes, because the interface is not intuitive to novice users. Therefore, mitigating errors should be an investment in human factor engineering methods from the beginning of the product development process to the training of the main users.

## 1. Introduction

To provide safe and high-quality care for patients, the medical care sector requires effective and well-designed devices. The project of these devices must consider the environment, the working pattern of the professional users, and the lifestyle of patients and caregivers [1].

For Donchin and Seagull [2], intensive care units (ICU) often use complex and error-prone devices because there are many patient data to be processed by health professionals simultaneously. Neonatal intensive care unit (NICU) is considered an environment of high complexity, with potential risk for serious adverse events to occur [3]. In this context, the neonatal incubator (NI) is an important medical equipment of life support, as the survival rate of premature newborns (NB) increases. In the 1950s, Silverman et al. [4] verified that 83.5% of the children maintained in the NI with higher temperatures (31.7°C) survived in comparison with 68.1% of the children kept in the NI with lower temperatures (28.9°C).

Antonucci et al. [5] affirm remarkable progress in the development of NIs, which are now technological devices. However, NI still needs improvements in several aspects. Ferris and Shepley [6] point the questions related to usability as one of them. They affirm that the improvements can support the medical team and parents in their interactions with the incubators, regarding programming, monitoring, and management of technology and ergonomic issues in visual and physical support for the baby. In addition, Maynard et al. [7] draw attention to the improvement on using this technology.

The human factors integration in the design process of medical devices is crucial to mitigate risks and to improve the patient security [8, 9]. Carayon et al. [10] point an approach to ergonomics and human factors as a central element of improving patient safety. Therefore, the incubators' interface must provide barriers to avoid errors of use and conflicting technological interactions that may cause injuries to the patient, considering the specific users, goals, and context, as recommended by the norm of usability focused on human-system interaction [11]. Thus, this study proposed to identify the main users, the frequent tasks, the major usability problems faced, and the context of use of a model of an NI in an NICU of a hospital in the Brazil's southeast region. It also aimed to know the problems faced by a novice user of an NI. The selected methods, heuristics analysis (HA), contextual inquiry, and usability testing (UT), were chosen to achieve these goals.

## 2. Materials and Methods

### 2.1. Objective of the Study

**Figure 1:**
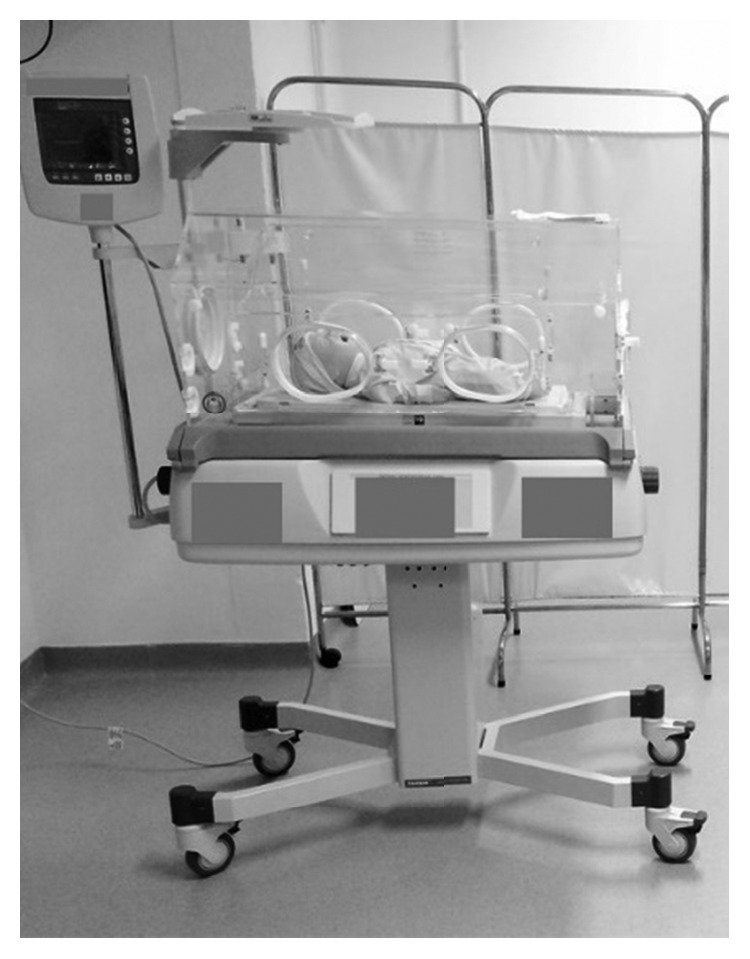
Neonatal incubator.

The objective of the research study was an NI made by a Brazilian company (Figure 1).

This model allowed temperature adjustments between 20°C to 37°C and 37.1°C to 39°C by pressing the >37°C key to the Air Mode, when the temperature inside the incubator rose gradually until the adjusted temperature. It also allowed temperature adjustments between 34°C to 37°C and 37.1°C to 38°C by pressing the >37°C key to the Skin Mode, when the temperature inside progressively increased until the NB temperature was equal to the suited temperature.

### 2.2. Study Design

#### 2.2.1. Ethics and Research Committee

The Ethics and Research Committee approved this research under the no. 1.506.90 on 04/19/2016.

#### 2.2.2. Research Stages

The study had four stages.

The first stage occurred with the manufacturer, which provided training and the equipment's documents to the researcher (the author RARC). This training guided the NI operation, disassembly, and assembly. Customers can have access to this training as soon as they purchase the equipment.

The second stage was performed at the Laboratory of Usability and Human Factors (LUFH) from the Federal University of Itajubá. The manufacturer provided an identical NI of the existing model in the NICU, which had its interface tested by five usability specialists through the HA method. This method allows finding existing usability problems [12] that compromise patient safety [13]. This research adopted the set of Nielsen–Shneiderman heuristics modified by Zhang et al. [13], which aimed to the evaluation of medical devices (Table 1).

The HA requires that three or more evaluators apply independently a set of usability heuristics to a product, identifying heuristics violations and grading their degree of severity (Table 2) [13].

According to Nielsen [15], five evaluators can find about 75% of the usability problems in the examined interface. In this research, five evaluators assessed the interface, performing 25 tasks (Table 3) to identify all the functions and to verify the usability problems location, the violated heuristic, and the degree of severity. Then, evaluations were consolidated into a single report. At the end of this stage, it was[[parms resize(1),pos(50,50),size(200,200),bgcol(156)]] and nursing coordination of the NICU, and the health team (doctors, nurses, nursing technicians, physiotherapists, speech therapists, and psychologists) to identify demands and the main users of the NI. Then, structured interviews were made with the nursing team (10 nurses and 34 technicians) to obtain the sociodemographic data. Some observations were performed to comprehend the activity on the unit and to learn the system variability. This stage allowed the identification of the representative users, the tasks frequently used in the NI, the context of use, and usability problems faced daily.

The fourth stage was executed at LUFH and evaluated the NI's interface through a usability test (UT) with novice users, to understand problems when using the equipment, which could prevent the correct use of this technology.

Five usability specialists formed a testing team, namely:

- A test leader (responsible for the research)
- Two observers, one of them being also an actor to represent the nursing technician of the surgical center that brings the NB to the test scenario
- An obstetrician nurse to represent the unit nurse
- A technician to operate the audio and video equipment

Fifteen volunteers, nursing students of the fourth year, with the following characteristics (Table 4) took part in the UT.

The test leader received them in a meeting room to guide the research and to collect the consent forms. The sociodemographic survey data were applied. Later, the Think Aloud technique was taught. The volunteers were guided to speak aloud while they were exploring the interface because the thought is not observable, and the observers needed to comprehend the volunteers' actions. For this purpose, they used a lapel microphone. They also received a training in Microsoft Power Point® 2016 to explain the basic functions of the equipment and level the group's knowledge.

**Figure 2:**
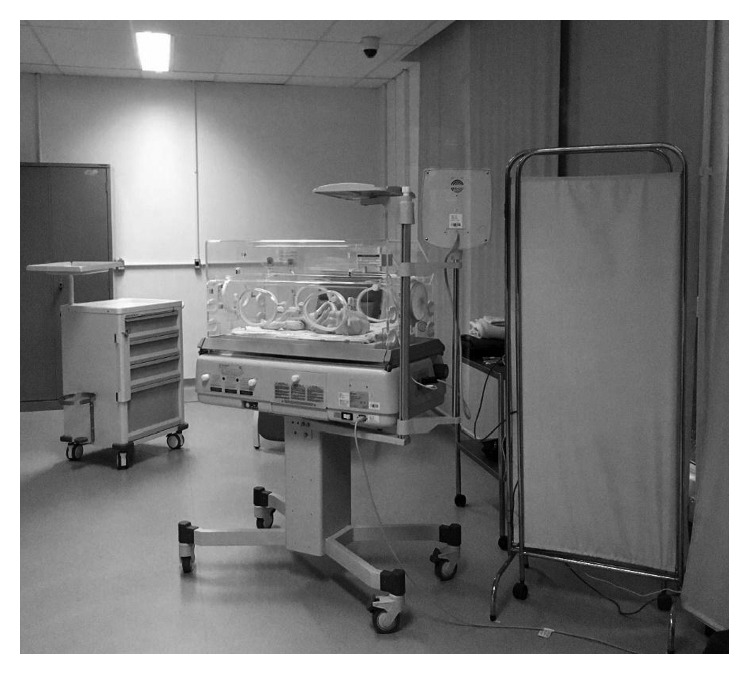
Simulated scenario for the UT of the NI.

Then, the volunteers went to the simulated scenario to participate in a realistic simulation of a nursery (Figure 2), where they would start their first day at work and they should prepare the NI to receive a premature baby whose mother was in the surgical center going through a caesarean session. The unit nurse received the volunteers and gave them the prescription of 16 tasks to do.

**Figure 3:**
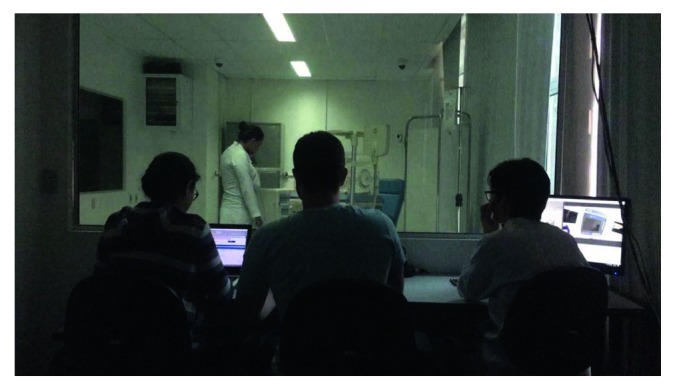
Observation room.

During the UT, the test leader remained with the two observers at the observation room with one-way glass and communicated with the volunteers via a microphone (Figure 3). After the execution of the eighth task, the NB (patient simulator) arrived at the nursery involved in a surgical field brought by the nursing technician with his medical record. The volunteer admitted the NB in the incubator and continued the other tasks. By the end of the test, the volunteers returned to the meeting room to answer another questionnaire to check their perception about the evaluated equipment. The tests were performed individually and lasted for about 1 h 30 min.

## 3. Results and Discussion

### 3.1. Training with the Manufacturer

The training with the manufacturer allowed the researcher to learn the operational mode of the NI. It was crucial to understand the manufacturer's point of view and how the information is transmitted through the training, as a guidance to operate the equipment in efficient, effective, and safe ways. However, the training is not always provided to the customers. Even when the professionals receive the training, it is usual to have team turnovers at NICUs, which cause knowledge to be lost over time. At the studied NICU, where 44 professionals are considered representative users, 28 (63.6%) did not receive training and 16 (36.4%) received some kind of training. Eleven of them (25.0%) were trained by more experienced colleagues and not by the manufacturer, 3 (6.8%) received training from clinical engineering and postgraduate courses, and the last 2 (4.6%) did not remember by whom they were trained. Thus, the interface should favor the correct use of its functions through good usability, regardless of the training. This is a challenge for the interface's developers because the context of use may vary, as pointed out by Maynard et al. [7]. These authors affirm that most of the neonatal health-care technologies are designed for high-income countries and are not available or are inadequate in places with fewer resources, preventing many NBs from receiving the appropriate standard of care.

The knowledge obtained during the training resulted in a list of tasks, which evaluators used during the HA. The manufacturer provided the instruction manual, which was used during this analysis.

### 3.2. Heuristic Analysis

**Figure 4:**
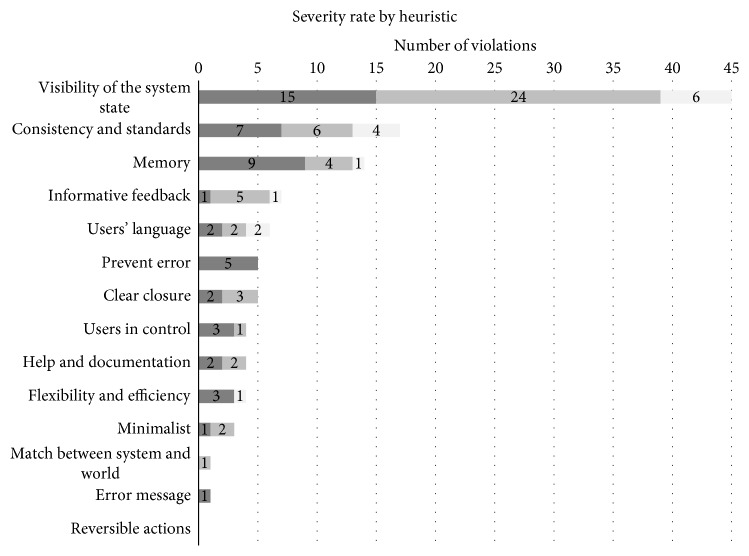
Plot of number of violations by heuristics and severity rate.

The HA led to the identification of 13 violations out of 14 heuristics listed by Zhang et al. [13] (Table 1). The Visibility of the System State heuristic presented the highest number of violations (45), showing that the user is sometimes uninformed about the system's state through feedback and information display. Besides, this heuristic had the highest number of problems with a severity rate 3, indicating a high priority for interface correction (Figure 4). The second heuristic with more violations was Consistency and Standards (17), which showed a lack of consistency between interface and manual and between graphical representations in the interface itself. The third more violated heuristic was Memory (14), which showed that the user needs to relearn the sequence to set a function because the interface does not provide enough and adequate information to reduce cognitive load.

**Figure 5:**
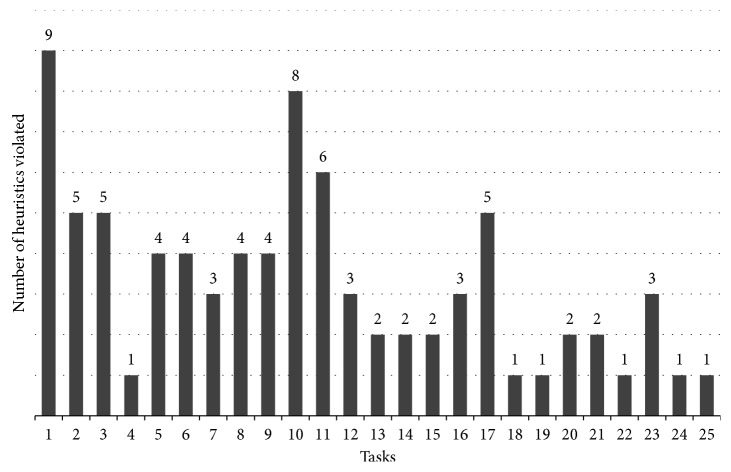
Plot of number of heuristics violated by analyzed task.

All the tasks violated at least one heuristic, and 69 usability problems were found. Because the same problem can violate more than one heuristic, the sum of violations was 82 points (Figure 5).

Figure 5 shows that task 1 (Turn on the neonatal incubator) was the one which violated more heuristics, followed by the tasks 10 (Set hour and date) and 11 (Lock the keyboard). This result points that the interface can receive several improvements by correcting identified problems, regardless of the severity. Nevertheless, the degree of severity is more important than the number of violated heuristics. The degree of severity must guide the manufacturer in the suitability of the interface, with respect to the priority of correction. No usability problems with a degree of severity 4 (meaning a catastrophic problem and imperative to correct) were found in this HA.

Table 5 highlights the main usability problems found with a degree of severity 3, which means a major problem with high priority to be corrected. So, it was possible to select the first sample containing 12 tasks out of 25 performed in the HA.

Among these 12 tasks, it is important to check whether they are all performed in real and relevant contexts to the end user.

### 3.3. NICU

#### 3.3.1. Representative User Profile

Among all the potential user groups, including the professional team, the patients' parents/relatives and the patient her/himself, we chose to analyze the more representative users, according to the NI manufacturer. Therefore, forty-four users who prepare and configure the equipment daily were assessed (Table 6), of whom 10 (22.7%) were nurses and 34 (77.3%) were nursing technicians. However, only nursing technicians really operate the NI because nursing's responsibility is to monitor the performance and guide the corrections if something is outside the desired parameters.

The users were mostly women (42, 95.5%), and the majority (28, 63.7%) were between 30 and 49 years of age. Thirty-two users (76.2%) worked over five years in the health-care area and 15 (34.1%) had worked over five years in the NICU. On the other hand, 29 (65.9%) had maximum four years working in the NICU. The hospital reported that, in the last year, there was a team turnover of 50%. Through a questionnaire, it was confirmed that 26 (59%) were in the NICU for one year and two months on average. This draws attention to the need for continued education in the unit, to maintain the team always updated in relation to the correct and safe mode of the equipment operation. This study did not investigate the reasons related to this high turnover rate among the staff members. Guse and Carvalho [16] affirm that the team turnover is multifactorial and complex. Thus, aiming to provide services with quality and that focus on patient's safety, the health-care team's continuous training is mandatory.

#### 3.3.2. Context of Use and Technological Interactions

The main context of use was the preparation of the NI, which happened on each patient's admission and every seven days when all beds had the equipment replaced by a sterilized one. At this moment, it was necessary to disassemble, to assemble, and to setup the parameters.

After the patient's admission, the NI had several technological devices associated with it, such as pulmonary ventilator, infusion pumps, parametric monitor, X-ray, and phototherapy equipment. They allow monitoring, diagnosis, and patient's treatment. However, it required a careful management by the team because it increased considerably the system complexity [17].

This system has technological interactions with the following equipment:

- X-ray unit: the device contains a space restriction because of the rotating shelf installed on the left rod of the equipment. This restriction can generate physical shock and causes significant noise for the NB. The noise is a direct cause of health, long-term hearing, and developmental issues. Sound levels are high in the NICU and can contribute to the harmful results often observed in premature NB [18].
- Phototherapy device: when next to the NI, this pedestal device has a rod that blocks the use of the fifth hatch. This inhibits the correct use of these hatches to remove contaminated material and makes it difficult to introduce medicine into the hood.
- Pulmonary ventilator: it has an interface through the trachea that goes through the iris sleeve. When it is not correctly closed, or when it opens by itself, the trachea can move and disconnect from the patient.
- Infusion pumps: usually four pumps are available for each bed. The teams put them through the tube inserts to reach the patient. In this case, careful handling is necessary for not losing the patient's venous accesses.
- Multiparameter monitor: the electrocardiogram, oximeter, and blood pressure cables also enter the NI through the tube inserts besides the iris sleeve, so it is necessary to check the connection to the patient.

The studied unit ventilator, the infusion pumps, and the multiparameter monitor have alarms that constantly trigger. According to PUL et al. [19], the number of alarms per hour per patient in a NICU is greater than that in the adult ICU. This may lead the professionals to an “alarm fatigue,” when the team is not aware of the alarms anymore due to the low rate of significant ones [20]. In this context, the alarm from the assessed NI distinguishes itself from the others by being higher and differentiated. This generates a quick inhibit action, but not necessarily a corrective one.

### 3.4. Tasks Performed at the NICU and Assessed in the HA and UT

Out of the 12 tasks (Table 4) with a degree of severity 3, eight were observed at the NICU. Thus, it was possible to select another sample to be analyzed and compared with the HA and UT results obtained from the volunteers' performance in a simulated environment.

#### 3.4.1. Turn on the Incubator

This task presented the highest number of heuristics violations (8) and usability problems (8) with a degree of severity 3. The existence of two master switches, being the one responsible to turn the panel on placed in NI's structure and not on the panel, led the specialists to conclude that this may represent an important problem to the novice user. As observed in the NICU, the users executed this task without difficulties because a senior professional provided guidance so that it did not represent an obstacle to put the equipment into operation because it was a routine.

However, this task performed in the UT with novice users showed that 100% could not conclude it and asked the nurse for help. They identified the posterior button to turn on and were not sure whether they have started the equipment or not, given that there was no sound or light validating the action (Feedback) neither any information about a second key to be triggered (Visibility/Documentation). All the participants searched for the panel power button in the NI's structure and not on its basis, pointing that there was no correspondence from the equipment to the user's world model (match between system and world). After being noticed about the second key's location, the users reported that the name “MASTER SWITCH” led them to believe that there was only one key (Users' Language/Consistency and Standards).

Five out of eight problems identified by the specialists and with a degree of severity 3 were also observed by the novice users. This confirms the need of an interface correction to make the tasks more intuitive and with no need of training. Nobody checked the manual to learn how to turn on the NI.

#### 3.4.2. Connect the Skin Sensor

All the NIs in the NICU allowed the skin mode that substantiates the need of this fixture. The connection of this sensor in the auxiliary sensor plug was not observed.

During the UT, 100% of the volunteers concluded the task. Sixty per cent connected the sensor based on the plug color because the sensor cable's label (patient sensor) differed from the connector's label (skin sensor), presenting the lack of consistence between the elements in the NI interface. This sensor connection was not quickly made; the users analyzed the text and colors for a while. It reaffirmed the heuristics violations of consistence, memory, and language, one of the two problems pointed out by the specialists in HA.

#### 3.4.3. Set the Temperature of the Skin Sensor to 36.4°C

After the skin sensor being placed on the NB's thigh, the task about setting the temperature of the skin sensor to 36.4°C started. The nursing technicians understood that the sensor would show the NB skin temperature but did not associate this with the NI operation mode, which had the air temperature controlled by the temperature of the patient's skin. In this case, there was not an operation difficulty, but an understanding one, which reduced the option by this mode, because the NI alarmed more often, as reported by the nursing team. During the observations, it was identified that only 43% of the NI was set to the Skin Mode.

After setting the skin temperature, the specialists noticed that the next step was not clear (visibility of the system state). Sometimes the screen returned automatically to the main screen, so the mode was not activated through the button “ON” (prevent error; memory) and the user was not warned about it (clear closure).

In the UT, the volunteers activated the button “ON” only in 40% of the 15 tests, just like the specialists. This makes evident the interface does not notify the task's conclusion, not guaranteeing its completion. Thus, the UT and the HA identified the same usability problems.

#### 3.4.4. Set Relative Humidity to 60%

During the first ten days of hospitalization, the humidity value is set at 80%. Then, it is downgraded to 60% on the remaining days of the patient's stay at the NICU. In the beginning of the observations, most NIs were not with the humidity turned on due to a flaw in the humidity sensor. According to the hospital's clinical engineering, the inhibition of the alarm without reading its message causes problems on identifying the lack of water in the reservoir, so the sensor burns. Regarding the use of humidification, 20% of the team complained about the condensation inside the hood, making it difficult to inspect visually the NB.

During the HA, the specialists had the same difficulty mentioned in the configuration of skin temperature. In the UT, because this task was the next after setting the skin temperature, 80% of the volunteers repeated the same steps followed previously to access the configuration screen, and then 53.3% activated this function, whereas 40% activated it in the previous task. This means that the users were familiar with the interface and realized necessary actions while exploring it.

#### 3.4.5. Verify the Date of Air Filter Replacement

At the NICU, the replacement date of the filter was not identified in the field destined for this purpose in the filter itself, which is inside a compartment in the back of the NI. On the other hand, it was on an adhesive label placed on the air filter compartment lid, keeping the date visible for control.

During the HA, one specialist (20%) searched for the date in the monitor and found only the change period (3 months). The others identified it in the filter, but they had difficulties to locate and read the date, so they had to remove the filter to read the field aimed at it (flexibility and efficiency).

Four volunteers (26.7%) did not find the air filter in the UT (visibility of the system state). Five volunteers (33.3%) pointed reading the replacement date and its location as a difficult action. The strategy adopted by the NICU to make the information more visible to reduce the cognitive load (Memory) is highlighted, showing a possibility to improve the interface making it more efficient.

#### 3.4.6. Locate Button for Adjusting Monitor Contrast

This function is rarely used because the NI's screen remains in the same configuration. There was an episode where the screen was too dark, and the professional found it difficult to locate the contrast adjustment button. According to the clinical engineering and the work order book, there were episodes when the equipment was sent for repair due to problems on the screen, and it was observed that the contrast button was just accidentally triggered.

In the HA, all the specialists searched for the button on the monitor and not on the anteroinferior side of the equipment. The same situation happened to 100% of the volunteers in the UT (match between system and world). It was necessary to explain to one of them (6.7%) that the contrast refers to the illumination of the screen. The term “contrast” may not be part of the users' language (users' Language).

#### 3.4.7. Identify Alarms

The alarms have an important role to alert the user about a parameter outside of the configured limits. It is unanimous among specialists, health professionals, and UT volunteers that the volume of NI's alarm is too loud. Therefore, it was necessary to inhibit it, and this did not allow identifying the reasons why it was triggered.

In the UT, despite the text alarm that kept flashing when the alarm sounded, the message was not read in 100% of the tests. After being asked about the reason why the alarm triggered, only five volunteers (33.3%) identified correctly through the text message. Among the others, five of them (33.3%) understood the alarm as a feedback for pressing the “TURN ON” button for the activated function. The connection with the feedback occurred because, in all tests, the alarm started immediately after the button was triggered. Four volunteers (26.7%) did not know the reason for the alarm. Only one (6.7%) made the connection between the text alarm and the alarm limits.

In the studied NICU, the NI's alarm sound was the loudest among all the equipment, which led to a quick inhibition without exactly correcting its cause. This became evident when there was no concern about the message indicating that the reservoir was empty. This means a hazard that could cause harm to the patient's health. In a survey conducted among the nursing team, 55% considered the alarm volume as a negative point and source of stress for the team and for the NB. An association is made with the excess alarms triggered on the Skin Mode. It led to the adoption of the Air Mode, in which the NI seemed to be more stable and with less alarms. The HA pointed the visibility problem as the cause of the alarm.

#### 3.4.8. Identify Air Mode and Skin Mode

The NI in the NICU must provide a suitable microclimate for the stabilization of the body temperature through the correct configuration of the parameters. The Skin Mode allows the adjustment of air temperature in function of the NB's body temperature, being the most suitable way for his/her development. In the NICU, this mode was activated in 43% of the NI in use. According to the nursing team, when the NI was in the Air Mode, the alarm triggered less frequently. They believed the Skin Mode could only monitor the NB's temperature, and if it changed, the alarm triggered. The NB's temperature was checked every 3 hours with axillary thermometer, and there was a difference on the temperature's value displayed by the skin sensor placed in the thigh of the NB and not in the abdomen in the liver's region as indicated by the manufacturer. Thus, the Air Mode was used preferentially on 57% of the NIs because there was more control over the air temperature and, therefore, fewer alarm episodes. The nursing team pointed the patient's hyperthermia as one concern. For this issue, the manufacturer recommends the use of the Skin Mode together with humidification.

In the UT, 14 volunteers (93.3%) indicated that the Air Mode showed the air temperature of the NI and the Skin Mode showed the NB's temperature. Only one volunteer (6.7%) did not know what the Air and Skin Modes were. No user related the Skin Mode with the air temperature changing in function of the NB's body temperature. These conclusions are aligned with the perception of the specialists that the interface does not allow to understand the meaning and the difference between the two modes (visibility).

### 3.5. The Tested Functions Related to Security

In conclusion, the tasks that may cause a hazard and bring harm to patients are mainly related to the following:

- The correct choice and activation of the Air or Skin Modes that may favor patient hyperthermia
- the alarms, regarding the volume and quick inhibition without reading the text alarm and without the corrective action
- the screen's adjustment contrast button, which can turn it off and lead to an unnecessarily repair, causing the reduction of a bed in the NICU
- the lack of visibility to check the air filter replacement date.

### 3.6. Actions Performed to Promote Good Practice

After the four stages of the present study, it was possible to execute actions to promote the efficient and safe use of this equipment.

#### 3.6.1. Together with the Manufacturer

A report was sent to the manufacturer according to the Brazilian standard ABNT NBR IEC 62366: 2010—health products—application of usability engineering to health products. The user profile, the intended conditions of use, the primary operations functions containing the frequently used functions and the safety-related functions, the usability specification containing the description of the intended scenario of use, and the worst-case scenario for validation were delivered in this document. The AH results were inserted in the item usability verification. The content of this report may guide interface's improvements of new versions.

#### 3.6.2. Together with the NICU

All the members of the nursing team received training, which addressed as the functional to the NI operating mode. It was possible to clarify doubts mainly about the alarms and the Air and Skin Modes and to guide the proper parameter configuration.

This work also contributed to the composing of six documents prepared by the NICU nursing coordination and named it as standard operational routines, aimed at guiding procedures that include the IN preparation and operation. These documents indicate the objective, the professionals involved, the responsible sector, and the description of the actions. They were divided into the following documents: (a) bed preparation for admission; (b) changing incubator air filter; (c) admission, incubator's humidification; (d) uterus use—nest repair; and (e) use of the incubator. These documents will be a reference for the ongoing training of experienced and novice users who will join the team.

## 4. Conclusions

This work enabled verifying the need of a detailed study using various methods from human factor engineering and the involvement of several process people to improve the efficiency and security of an interface. The understanding of different points of view about the product, namely, the manufacturer, the usability experts, the normative, the clinical engineering, the experienced users, the novice users, and the environment where it is placed, reveals that the systemic vision is the one that brings the best result and assertiveness to mitigate risks for the patient. The HA, contextual investigation, and UT methods formed an efficient set to achieve such assertiveness.

Nurses and technicians are the main users of the IN; the last ones are those who operate the equipment daily. Eight frequently used tasks were highlighted to be performed in the UT, and the main faced problems involve the alarms and the configuration of the Air and Skin Modes. The main context of use is the admission of the NB and the replacement of the equipment when the NI needs preparation and configuration. The execution of the UT with novice users could lead to verify that the interface is not intuitive for the eight tasks analyzed. The IN instruction manual was not used and that training is necessary to ensure the correct use of functions. The two following tasks were in evidence: the alarms identification, which was correctly performed by only 6.7% of the novice users; and the identification of Air and Skin Modes, which revealed that neither specialists nor senior and new users understood the difference between the two modes. These abovementioned conclusions point that the work to mitigate errors must begin in the product development process, inserting human factors engineering methods, going through the academic training of health professionals involving technology as a school subject, and reaching the senior user through training for the functions that may cause a hazard to the patient.

## Figures and Tables

**Table 1:** Zhang et al.'s 14 heuristics list.

Heuristic	Explanation
(1) Consistency and standards	Users should not have to wonder whether different words, situations, or actions mean the same thing. Standards and conventions in product design should be followed
(2) Visibility of the system state	Users should be informed about what is going on with the system through appropriate feedback and display of information
(3) Match between system and world	The image of the system perceived by users should match the model the users have about the system: actions and objects on the system should match actions and objects familiar to the users
(4) Minimalist	Any extraneous information is a distraction and a slowdown
(5) Memory	Users should not be required to memorize a lot of information to carry out tasks. Memory load reduces users' capacity to carry out the main tasks
(6) Informative feedback	Users should be given prompt and informative feedback about their actions
(7) Flexibility and efficiency	Users always learn, and users are always different. Give users the flexibility of creating customization and shortcuts to accelerate their performance
(8) Error message	The messages should be informative enough such that users can understand the nature of errors, learn from errors, and recover from them
(9) Prevent error	It is always better to design interfaces that prevent errors from happening in the first place
(10) Clear closure	Every task has a beginning and an end. Users should be clearly notified about the completion of a task
(11) Reversible actions	Users should be allowed to recover from errors. Reversible actions also encourage exploratory learning
(12) Users' language	The language should be always presented in a form understandable for the intended users
(13) Users in control	Do not give users the impression that they are controlled by the systems
(14) Help and documentation	Always provide help when needed

Source: reproduced from Bernardes et al. [14].

**Table 2:** Severity rating scale.

(0) Not a usability problem at all
(1) Cosmetic problem only. Need not be fixed unless extra time is available
(2) Minor usability problem. Fixing this should be given low priority
(3) Major usability problem. Important to fix. Should be given high priority
(4) Usability catastrophe. Imperative to fix this before product can be released

Source: adapted from Zhang et al. [13].

**Table 3:** Tasks list.

	Tasks
(1)	Turn on the neonatal incubator
(2)	Connect the skin sensor
(3)	Connect the humidity sensor
(4)	Connect the auxiliary sensor
(5)	Set the skin sensor temperature to 36.4°C
(6)	Set humidity to 60%
(7)	Select the trend graphic of air temperature
(8)	Verify the trend graphic of skin temperature
(9)	Verify the trend graphic of humidity
(10)	Set hour and date
(11)	Lock the keyboard
(12)	Write on a paper the hour and date, and the humidity and the temperatures of Air mode, Skin Mode, and Auxiliary mode
(13)	Locate the water reservoir compartment
(14)	Remove the water reservoir from the compartment
(15)	Locate the air filter
(16)	Check air filter replacement date
(17)	Adjust trend graphical display in 8 h
(18)	Identify input for limited oxygen hose
(19)	Identify the compartment for the X-ray plate
(20)	Locate the button for contrast adjustment of monitor
(21)	Locate connector for computer cable
(22)	Raise the bed at the head height and then at the feet height
(23)	Identify alarms
(24)	Read the patient's identification card
(25)	Identify the Air Mode and Skin Mode

Source: author.

**Table 4:** Profile of UT volunteers sample.

Gender (%)	Age (%)	Laterality (%)	Average height (m)
Female: 86.7	Up to 29 years: 93.3	Right-handed: 86.7	1.65
Male: 13.3	From 30 to 39 years old: 6.7	Left-handed: 13.3	

**Table 5:** Problems with a degree of severity 3.

Task	Problem description	Violated heuristics
(1) Turn on the neonatal incubator	The incubator has two keys, named MASTER SWITCH, one on its back and the other on its anteroinferior side, which can raise questions to turn on the equipment	(12) Users' language
	The back MASTER SWITCH has visibility problems related to the power icon that is in the same color of the button. The user can turn the equipment off or on because the icon is not visible	(2) Visibility of the system state
	There is no indication that the back MASTER SWITCH is the first to be pushed. The user can press only the anteroinferior key that turns on the panel but not the incubator	(2) Visibility of the system state
		(5) Memory
		(14) Help and documentation
	There is no feedback when the MASTER SWITCH is turned on. The button clicks but it does not turn on any light or emit any sound, indicating that the action was successfully executed	(6) Informative feedback
	In the manual, the button on the anteroinferior side is identified as CONTROL PANEL SWITCH. However, in the equipment, it is written MASTER SWITCH, which can confuse the user	(1) Consistency and standards
	There are two buttons to turn on the incubator. If the user does not press them, the incubator will not start its normal operation	(5) Memory
	If the user does the incorrect procedure to turn on the incubator, there is no information/message to indicate it	(8) Error message
	The information in the power cable is insufficient for the user to know the proper equipment voltage	(5) Memory
		(9) Prevent error

(2) Connect the skin sensor	On the sensor cable's label, it is written PATIENT SENSOR, and in the power plug it is written as SKIN SENSOR. The user needs to think if one matches the other. It can raise questions about it	(1) Consistency and standards
		(5) Memory
		(12) Users' language
	It is possible to connect the AUXILIARY SENSOR cable to the SKIN SENSOR power plug	(9) Prevent error

(3) Connect the humidity sensor	The humidity cable that is connected to the oxygen calibration cells indicates that this cable is from oxygen and not from humidity, which can generate doubts for the user whether to connect it or not	(1) Consistency and standards

(5) Set the skin sensor temperature to 36.4°C	It is not clear whether it is necessary to press ON for the SKIN SENSOR to start working after temperature configuration because the screen automatically returns to the main screen after a while	(2) Visibility of the system state
		(5) Memory
		(9) Prevent error
	After configuring the temperature, there is no option to confirm the action	(10) Clear closure

(6) Set humidity at 60%	It is not clear whether it is necessary to press ON for the humidity to start working because the screen automatically returns to the main screen after a while	(9) Prevent error
	When setting the humidity value, there is no indication to activate the function, if it was not done yet	(5) Memory
		(9) Prevent error

(10) Set hour and date	The manual guides you to click on the MENU icon, but it is not found	(1) Consistency and standards
		(13) Users in control
		(14) Help and documentation
	When pressing the icon with a multisheet paper drawing corresponding to the MENU (in the lower right corner), it is not very clear what should be done on the next screen	(2) Visibility of the system state
	Difficulty on finding and accessing the clock option. The sequence of steps is unclear and unguided	(1) Consistency and standards
		(2) Visibility of the system state
		(5) Memory
		(10) Clear closure

(11) Lock the keyboard	Difficulty on performing the sequence of steps to lock the keyboard. The sequence is very confusing, long, and unguided	(1) Consistency and standards
		(2) Visibility of the system state
		(4) Minimalist
		(5) Memory
		(7) Flexibility and efficiency
	The MENU key must be pressed twice consecutively to access the screen to lock the keyboard. The user must explore the interface until he finds out this need and may not be able to locate the menu that allows access to the function	(2) Visibility of the system state
	One of the evaluators did not identify the steps to lock the keyboard	(2) Visibility of the system state
		(13) Users in control
(16) Check air filter replacement date	Difficulty in finding the replacement date of the air filter, which is in a very small space on the filter itself inside a compartment behind the IN	(2) Visibility of the system state
		(5) Memory
		(7) Flexibility and efficiency

(17) Adjust trend graphical display in 8h	The task was only performed after exhaustive attempts. The system is not intuitive in relation to the button that displays the MENU function. In this function, it is possible to notice that there is more than one screen indicating that, in each activation, a new screen will open. However, only this information is not enough for a novice user to recognize the functionality and the need to press the button twice. The system presents no other alternative for the user to recognize the steps to execute the action	(2) Visibility of the system state
		(7) Flexibility and efficiency
		(13) Users in control

(20) Find the button for adjusting monitor contrast	The evaluator looked for the contrast button on the monitor, not on the anteroinferior side of the equipment, where the button is located	(3) Match between system and world

(23) Identify alarms	The alarm sounds quite high which can cause the user to silence it quickly without worrying about why it is alarming	(2) Visibility of the system state

(25) Identify air Mode and skin mode	The user who has not been trained may not identify the Air Mode and Skin Mode meanings and why the incubator does not allow activating both modes at the same time	(2) Visibility of the system state

Source: author.

**Table 6:** NICU's user profile.

Nursing team						
Gender (%)	Age (%)	Laterality (%)	Average height (m)	Shift (%)	Working time in the health-care area (%)	Working time in the NICU (%)
Female: 95.5	Up to 29 years: 36.4	Right-handed: 94.9	1.63	Daytime: 47.7	Less than 1 year: 0	Less than 1 year: 13.6
	From 30 to 39 years: 52.3				From 1 to 4 years: 23.8	From 1 to 4 years: 52.3
Male: 4.5	From 40 to 49 years: 11.4	Left-handed: 5.1		Night: 52.3	From 5 to 10 years: 50	From 5 to 10 years: 25
					Over 10 years: 26.2	Over 10 years: 9.1

## Data Availability

The data used to support the findings of this study are included within the supplementary information files.

## Supplementary Materials

Supplementary 1 Figure 4: it shows the number of violated heuristics according to the severity rate, detected during the Heuristic Analysis of the neonatal incubator.

Supplementary 2 Figure 5: it shows the number of both violated heuristics and usability problems of each described task, detected during the Heuristic Analysis of the neonatal incubator.
