# Supplementary material for: Applying Human Factors Engineering Methods for Risk Assessment of a Neonatal Incubator
Source: J Healthc Eng. 2019 Jan 6;2019:8589727. doi: 10.1155/2019/8589727 (PMC6339723; doi:10.1155/2019/8589727)
Supplement: Supplementary 1 — Figure 4: it shows the number of violated heuristics according to the severity rate, detected during the Heuristic Analysis of the neonatal incubator. [file 8589727.f1.pdf]

	Zhang's Heuristics	Severity rate					Number of violations
		4	3	2	1	0	
1	Visibility of the system state	0	15	24	6	0	**45**
2	Consistency and Standards	0	7	6	4	0	**17**
3	Memory	0	9	4	1	0	**14**
4	Informative Feedback	0	1	5	1	0	**7**
5	Users' Language	0	2	2	2	0	**6**
6	Prevent Error	0	5	0	0	0	**5**
7	Clear Closure	0	2	3	0	0	**5**
8	Users in Control	0	3	1	0	0	**4**
9	Help and documentation	0	2	2	0	0	**4**
10	Flexibility and efficiency	0	3	0	1	0	**4**
11	Minimalist	0	1	2	0	0	**3**
12	Match between system and world	0	0	1	0	0	**1**
13	Error Message	0	1	0	0	0	**1**
14	Reversible actions	0	0	0	0	0	**0**
