# Supplementary material for: Applying Human Factors Engineering Methods for Risk Assessment of a Neonatal Incubator
Source: J Healthc Eng. 2019 Jan 6;2019:8589727. doi: 10.1155/2019/8589727 (PMC6339723; doi:10.1155/2019/8589727)
Supplement: Supplementary 2 — Figure 5: it shows the number of both violated heuristics and usability problems of each described task, detected during the Heuristic Analysis of the neonatal incubator. [file 8589727.f2.pdf]

	Task	heuristics violated	usability problems
**1**	Turn on the neonatal incubator	9	13
**2**	Connect the skin sensor	5	3
**3**	Connect the humidity sensor	5	5
**4**	Connect the auxiliary sensor	1	1
**5**	Set the skin sensor temperature in 36.4°C	4	2
**6**	Set humidity in 60%	4	4
**7**	Select the trend graphic of air temperature	3	2
**8**	Verify the trend graphic of skin temperature	4	3
**9**	Verify the trend graphic of humidity	4	4
**10**	Set hour and date	8	6
**11**	Lock the keyboard	6	3
**12**	Write on a paper the hour and date, the humidity and the temperatures of Air Mode, Skin Mode and Auxiliary mode	3	5
**13**	Locate the water reservoir compartment	2	1
**14**	Remove the water reservoir from the compartment	2	2
**15**	Locate the air filter	2	1
**16**	Check air filter replacement date	3	2
**17**	Adjust trend graphical display in 8h	5	2
**18**	Identify input for limited oxygen hose	1	1
**19**	Identify the compartment for the x-ray plate	1	1
**20**	Locate the button for contrast adjustment of monitor	2	2
**21**	Locate connector for computer cable	2	1
**22**	Raise the bed at the head height and then at feet height	1	1
**23**	Identify alarms	3	2
**24**	Read the patient’s identification card	1	1
**25**	Identify Air Mode and Skin Mode	1	1

**82**	**69**
